# Modeling hallmark pathology using motor neurons derived from the family and sporadic amyotrophic lateral sclerosis patient-specific iPS cells

**DOI:** 10.1186/s13287-018-1048-1

**Published:** 2018-11-15

**Authors:** Xuejiao Sun, Jianyuan Song, Hailong Huang, Hong Chen, Kun Qian

**Affiliations:** 1 Department of Rehabilitation Medicine, Tongji Hospital, Tongji Medical College, Huazhong University of Science and Technology, Jiefang Avenue 1095, Wuhan, 430030 China; 2 Reproductive Medicine Center, Tongji Hospital, Tongji Medicine College, Huazhong University of Science and Technology, Jiefang Avenue 1095, Wuhan, 430030 China

**Keywords:** Amyotrophic lateral sclerosis, TDP-43, Induced pluripotent cells, Neurofilament, Mitochondria

## Abstract

**Background:**

Amyotrophic lateral sclerosis (ALS) represents a devastating, progressive, heterogeneous, and the most common motor neuron (MN) disease. To date, no cure has been available for the condition. Studies with transgenic mice have yielded significant results that help us understand the underlying mechanisms of ALS. Nonetheless, none of more than 30 large clinical trials over the past 20 years proved successful, which led some researchers to challenge the validity of the preclinical models.

**Methods:**

Human-induced pluripotent cells (iPSCs) were established by introducing Sendai virus into fibroblast cells. We established TDP-43 HES by inserting CAG-TDP43 (G298S) cassette or the CAG-EGFP cassette into PPP1R12C-locus of human embryonic stem cells (ESC, H9) by TALEN-mediated homologous recombination. iPSCs or HESC were differentiated to motor neurons and non-motor neuron as control. Relevant biomarkers were detected in different differentiated stages. TDP-43 aggregates, neurofilament, and mitochondria analyses were performed.

**Results:**

In this study, using iPSCs-derived human MN from an ALS patient with a *TDP43* G298S mutation and two sporadic ALS patients, we showed that both sporadic and familial ALS were characterized by TDP-43 aggregates in the surviving MN. Significantly higher neurofilament (NF) inclusion was also found in ALS MN compared with wild-type (WT) GM15 controls (*P* < 0.05). The neurite mitochondria density was significantly lower in ALS MN than that in the control MNs. Transgenesis of *TDP-43* G298S into AAVS locus in human embryonic stem cells reproduced phenotype of patient-derived G289S MN. By challenging MNs with a proteasome inhibitor, we found that MNs were more vulnerable to MG132, with some accompanying phenotype changes, such as *TDP43* translocation, NF inclusion, mitochondria distribution impairment, and activation of caspase3.

**Conclusions:**

Our results suggested that changes in TDP43 protein, NF inclusion, and distribution impairment of mitochondria are common early pathology both in familial and sporadic ALS. These findings will help us gain insight into the pathogenesis of the condition and screen relevant drugs for the disease.

**Electronic supplementary material:**

The online version of this article (10.1186/s13287-018-1048-1) contains supplementary material, which is available to authorized users.

## Background

Amyotrophic lateral sclerosis (ALS) is a devastating, progressive, heterogeneous, and the most common motor neuron (MN) disease [1]. Incidence of ALS stands at 1–2 per 10,000, and the risk of ALS is estimated to be 1/600–1/2000 [2]. Approximately 90% of all cases of ALS are of sporadic nature, defined as the absence of family history of the disease, and their causes remain enigmatic [3]. About 10% of ALS cases are of familial nature and are associated with mutations in several genes such as *C9orf72*, *SOD1*, *TARDBP*, *FUS*, and *ANG*. *TARDBP* gene mutations account for about 4% of familial ALS. The protein encoded by TAR DNA-binding protein 43 (TDP-43) was a major component of the histopathological hallmark of degenerating neurons in most sporadic and familial ALS cases [4]. The disease is fatal within 2–5 years after onset, mostly because of respiratory failure. So far, there is no cure for the condition [5].

Work with transgenic mice has led to important advances in the understanding of mechanisms of ALS. Since 20- to 40-fold of mutant protein is needed to produce a phenotype in mice, artificial phenotypes might result [6]. Genetic and anatomical variations exist between rodents and human beings. Furthermore, most ALS are sporadic and it is difficult to create animal models of the condition [7]. So it is not a surprise that none of more than 30 large clinical trials in the past 20 years were successful, leading some researchers to question the validity of the preclinical models [8]. However, recent success in reprogramming somatic cells to induced pluripotent stem cells and our newly developed high-purity MN differentiation system provide a novel approach for the study of ALS. The experimental findings can help screen drugs and establish models of human cells that carry natural mutation or cells from sporadic cases. However, before any trial on candidate drugs for ALS begins, we have to know whether the abnormal cellular and molecular phenotypes of ALS can be recapitulated in vitro. In this study, we reported the phenotypes of cellular pathology in iPSC-derived human MN from an ALS patient carrying the G298S mutation and two sporadic ALS patients.

## Materials and methods

### Human iPSCs

The fibroblast information of GM15, G298S TDP43, and two sporadic was provided in Additional file 1: Table S1. Supplemental data. Informed consents were obtained before skin biopsy. The fibroblasts were reprogrammed using Sendai virus as previously described [9]. To obtain virus-free cell lines, the iPSCs were stored at 39 °C for 7 days. IMR-90 cells were obtained from ATCC CCL-188.

### Teratoma formation

Human ESCs and iPSCs were subcutaneously injected into the back of mice with severe combined immunodeficient (SCID) mice (Jackson Laboratory, Bar Harbor, ME, http://www.jax.org). Two months after the development of tumors, mice were perfused with 4% paraformaldehyde and teratomas were HE-stained.

### MN and non-MN differentiation

Human iPSCs or ESCs were first differentiated to neuroepithelia for 7 days in a neural medium consisting of Dulbecco’s modified Eagle’s medium/Ham’s F-12 medium (Gibco, Grand Island, NY, http://www.invitrogen.com), non-essential amino acids, and N2 supplement in the presence of 2 μM SB431542, 3 μM CHIR99021 and 300 nM LDN193189 (all from Stemgent). At day 8, for MN induction, the neuroepithelia were treated with 0.1 μM retinoic acid (RA) and 0.5 μM purmorphamine (both from Stemgent) for 7 days. 0.5 μM cyclopamine was added in place of purmorphamine to generate non-MNs. At day 14, both the rosettes-like MN and non-MN progenitors were isolated with a pipette and expanded as floating clusters in suspension in the same respective medium but without SB431542, LDN193189, and CHIR99021 for an additional 7 days. The immature MN and non-MN clusters at day 21 were dissociated into individual cells or plated neurospheres onto laminin substrate in the presence of 0.1 μM compound E, a notch inhibitor to block cell proliferation for 10 days to produce synchronized post-mitotic MN and non-MN populations. The MNs or non-MNs were assayed from day 1 to day 10 after day 21 plating.

### Immunocytochemistry and cell counting

The primary antibodies are presented in Additional file 2: Table S2. ImageJ was used for cell counting (W. Rasband, NIMH, Bethesda, MD, USA). Neurofilament aggregates were defined as a distinct, inclusion-like focal accumulation of immunoreactive products with the intensity of the inclusion being three times higher than that of its surroundings [10]. At least 500 neurites were counted in each group.

### Immuno-electron microscopy

Cultures were fixed in 2.0% paraformaldehyde in 0.1 M cacodylate buffer (pH 7.4), 2.5% glutaraldehyde for 30 min for regular EM. Cultures were fixed in 4% paraformaldehyde in 0.1 M cacodylate buffer (pH 7.4), 0.1% glutaraldehyde for immuno-EM. The fixed cultures were rinsed with PBS, and residual aldehyde was inactivated by incubation for 10 min in 0.1% NaBH4 in 0.1 M PB. They were then permeabilized in 0.2% Triton-X-100 and incubated in AURION Blocking Solution (Aurion, Waganingen, Netherlands, http://www.aurion.nl source) for 30 min before incubation with TDP43 antibody (1:200) at 4 °C overnight. After rinsing six times, the cultures were incubated with the Ultra Small gold-conjugated secondary antibody overnight at 4 °C. The samples were then washed and post-fixed in 2% glutaraldehyde in 0.1 M PB and then subjected to regular embedding and EM processing. In order to increase the contrast of citrate, some thin sections were stained with uranyl acetate. For better visualization of immune-gold particles some sections were not stained. The samples were examined under a Philips 120 electron microscope.

### Western blotting

Cells were lysed using a buffer consisting of 62.5 mMTris-HCl at pH 6.8, 20% glycerol, 2% SDS, 2 mM DTT, 100 μM PMSF, and protease inhibitor cocktail. Lysates were resolved by SDS-PAGE, and Western blotting was carried out using horseradish peroxidase-conjugated IgG as a secondary antibody and the ECL system (Thermo Scientific, Rockford, IL, http://www.piercenet.com) for detection.

### Analysis of mitochondria density

For density quantification, mitochondria were labeled for 15 min at 37 °C by using 50 nM MitoTracker Orange CM-H2 TMRos (Life Technologies, USA). Then, the cells were fixed in 4% PFA made from culture medium. The length of entire neuritis stained with NF 200 antibody was measured. Images were analyzed using Image J (NIH) software to determine the number of mitochondria within the neurite. One hundred fifty neurons were randomly selected for analysis. The number of the mitochondria on a 50-μm segment from cell body was counted, and the sum of the mitochondria was divided by neuritis length.

### Live cell imaging

For labeling of NF-inclusion, 0.5 μg/ml doxycycline was added into the MN medium to induce NFL-GFP fusion protein expression 24 h before assay. MN mitochondria were then labeled for 15 min at 37 °C with 50 nM MitoTracker Orange CM-H2TMRos (Life Technologies, USA). MNs were washed and then replaced in complemented medium. Cells were observed at least 1 h after the washout of MitoTracker. Time-lapse recordings of movement of mitochondrial and NF inclusions were acquired every 5 s for 10 min by using a Nikon Biostation IM, in which the cells were maintained at 37 °C, in 5% CO_2_ in a sealed observation chamber during image acquisition.

### Gene editing of iPSCs and hESCs by TALEN-mediated homologous recombination

We successfully induced constitutive and inducible expression of transgenes in human ESCs and iPSCs by using TALEN-mediated homologous recombination. CAG-GFP cassette of the donor plasmid AAV-CAGGS-EGFP (Addgene, Cambridge, MA, http://www.addgene.org) was replaced by CAG-MCS cassette by SpeIand MIuIdouble digestion. To elicit the expression of G298S TDP43, the cDNA was inserted into the SalIand MIuIsites of donor plasmid. For the expression of Tet-On 3G, Tet-On 3G sequence was inserted into the MCS by XhoIand AscIdigestion. In order to construct Tet-inducible expression systems, pTRE3G-SV40-polyA cassette (pTRE3G, Clontech, Mountain View, CA, http://www.clontech.com/) was inserted into the SpeIsite of donor plasmid. For expression NFL-EGFP, the sequence was inserted into the SalIand MIuIsites of the inducible donor plasmid. TALENs pairs targeting the AAVS1 locus was designed as previously described [11]. By using Joung lab REAL assembly TALEN kit (Addgene, Cambridge, MA, http://www.addgene.org/), TALEN repeat arrays were constructed using standard restriction digestion and ligation reactions. TALEN activity was assayed via surveyor nuclease (Transgenomic, Omaha, NE, http://www.transgenomic.com/). Human iPSCs or ESCs were cultured in 1 μM Rho Kinase (ROCK)-inhibitor (Calbiochem, USA) 24 h prior to electroporation. Cells (1 × 10^7^) were electroporated with 40 μg of donor plasmids and 5 μg of each TALEN-encoding plasmid (Bio-Rad, Hercules, CA: 250 V, 500 μF, 4 mm cuvettes). Cells were subsequently plated onto MEF feeder layers in the MEF-conditioned medium supplemented with ROCK inhibitor for the first 24 h. Individual colonies were selected and expanded after puromycin selection (1 μg/ml) for 10 to 14 days. The correct cell clones were identified by PCR. The following primers are used: Forward: 5′ACCAACGCCGACGGTATCAG3′; Reverse: 5′CAGACCCTTGCCCTGGTGGT3′.

### Statistical analysis

For comparison between two groups, *t* test was used. Comparison between different groups and different time points was made using two-way ANOVA. In all cases, differences were considered to be statistically significant when *P* < 0.05.

## Results

### Establishment of virus-free iPS cell line

**Fig. 1:**
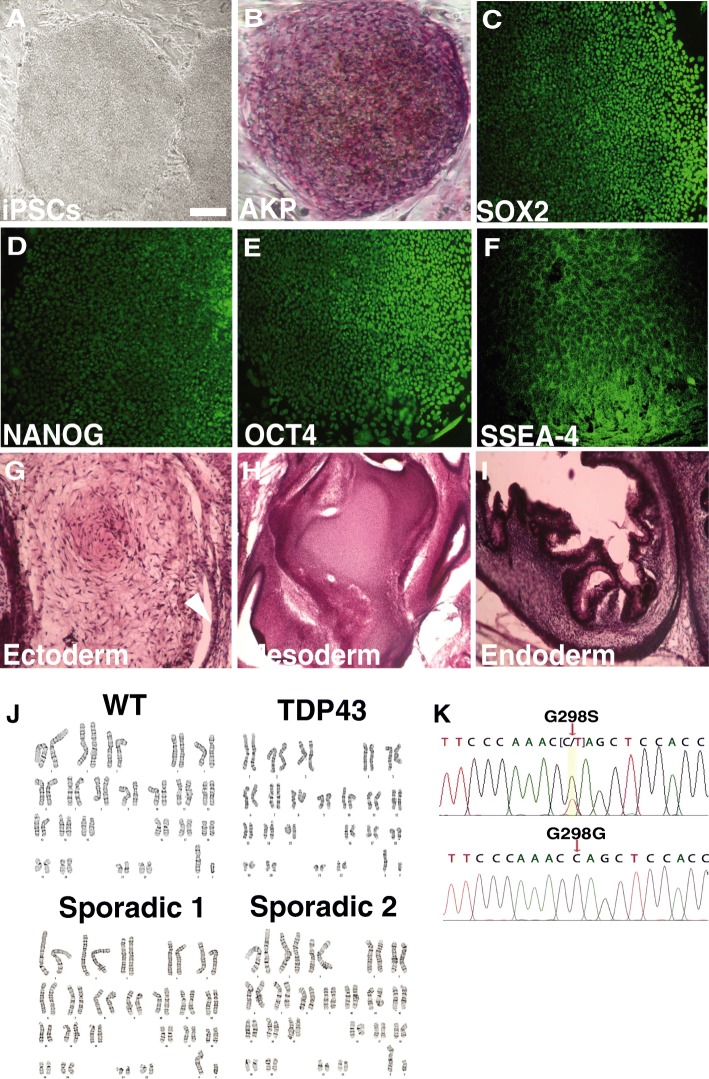
iPSC generation. **a** Contrast image of iPSC colonies generated by Sendai virus. Scale bar = 50 μm. **b** Alkaline phosphatase (AKP) staining of iPSC. **c**–**f** Immunofluorescent image of SOX2 (**c**), NANOG (**d**), OCT4 (**e**), and SSEA-4 (**f**) expression in ALS iPSCs. **g**–**i** HE staining of teratoma from ALS iPSCs showing gut epithelia (endoderm), cartilage (mesoderm), and epiderm (ectoderm) like structures. **j** Karyotyping of iPSCs. (**k**) DNA sequencing showing heterozygous nucleotides (C/T) in TDP43, but not sporadic and WT iPSCs

GM15 (as WT control), TDP43-1, TDP43-2, SPOR-1, and SPOR-2 iPSCs were established by introducing Sendai virus into fibroblast cells [9]. All iPSCs were virus-free after treatment according to the protocol reported by Ban et al. [9]. All iPSCs possessed comparable morphology mimicking that of human embryonic stem cell lines (Fig. 1a), and expressed the pluripotency markers: AKP, SOX2, NANOG, OCT4, and SSEA4 (Fig. 1b–f). The pluripotency was also confirmed by teratoma formation in vivo (Fig. 1g–i).The karyotypes stayed normal in all clones for more than 40 passages (Fig. 1j). The *TDP-43* G298S mutation was confirmed by sequencing the exon 6 of *TDP-43* (Fig. 1k).

### iPSC from ALS patients differentiated to motor neurons with high efficiency

**Fig. 2:**
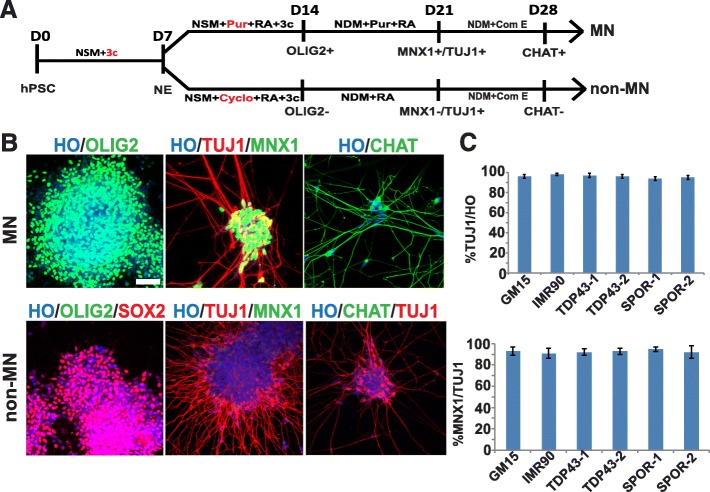
Neuron differentiation of iPSCs. **a** Schematic protocol for MN and non-MN differentiation. 3c, 3 small molecular compounds; Pur, purmorphamine; Cyclo, cyclopamine. **b** Immunofluorescent images of OLIG2^+^ MN progenitors, MNX1^+^postmitotic MNs, CHAT^+^ maturing MNs, SOX2^+^ progenitors, MNX1^−^ and CHAT^−^ spinal non-MNs. **c** Quantification of TUJ^+^ neuronal population among total Hoechst-labeled (HO) cells and MNX1^+^ MNs among neurons. Scale bar = 50 μm

We generated spinal cord motor neurons (MNs) by using a chemical protocol described in the “Materials and methods” section (Fig. 2a). The GM15, *TDP43*-1, *TDP43*-2, SPOR-1, and SPOR-2 iPSCs can differentiate into OLIG2^+^cells, HB9^+^/TuJ1^+^, and CHAT^+^ motorneurons with high efficiency (Fig. 2b). Seven days after the neurospheres were plated onto a plate, more than 90% were TuJ1-positive cells or HB9^+^ (CHAT^+^) motor neurons (Fig. 2c). There was no significant difference in the efficiency of MN generation among the groups (*P* > 0.05). Meanwhile, we used non-MNs as controls to confirm that the pathology is specific for MNs.

### Small TDP43 aggregates and TDP43 translocation were found in G298S *TDP43* and sporadic MNs by EM

**Fig. 3:**
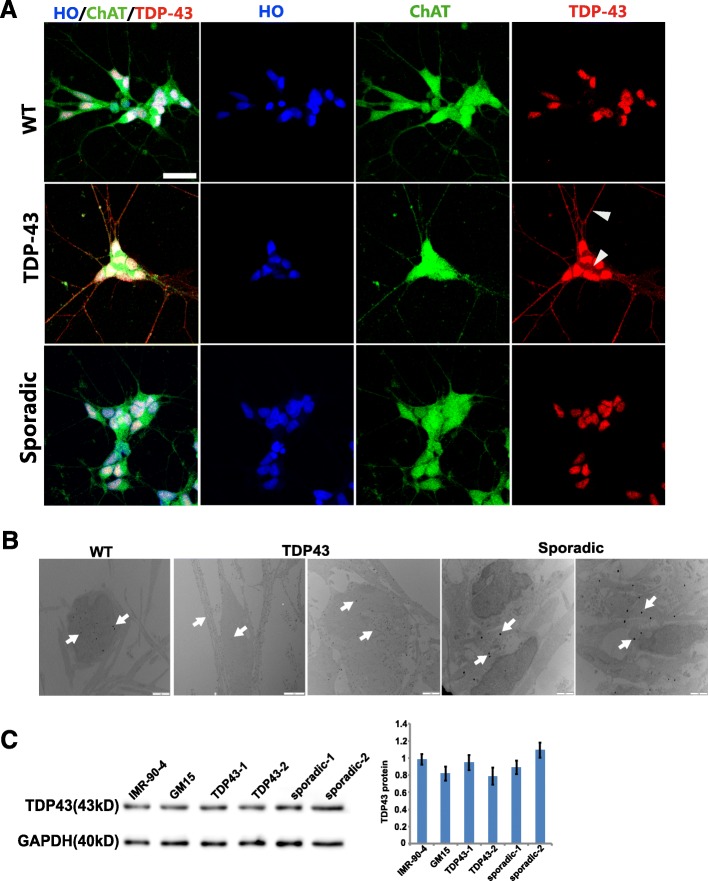
TDP43 expression and aggregation in iPSC-derived neurons. **a** Immunofluorescent image of TDP43 expression in WT, TDP43, and sporadic MN. Translocation of TDP43 was found in G298S TDP43 MN. Scale bar = 50 μm. **b** TDP43 immuno-EM in neurites, cytoplasm and nuclei of MN cultures. TDP43 MN had a lot of small gold particles in the neurites and cytoplasm, while sporadic MNs had gold particle clusters. Arrows = clusters of gold particles. No contrast staining for ALS MNs for better view of fine gold particles. Scale bar = 2 μm. **c** Representative Western blots and relative TDP43 expression levels (to GAPDH) in MNs

Normal TDP-43 is a nuclear protein. Pathological TDP-43, however, is redistributed and sequestered as protein aggregates in neuronal nuclei, perikarya, and neurites in the spinal cord and brain of sporadic and non-*SOD1* mutant familial ALS patients, and is supposed to impair the cell through multiple mechanisms [12, 13]. Our immunocytochemical analysis revealed that TDP-43 was mainly localized in the nucleus in the sporadic MNs and WT control MNs, whereas in very few *TDP-43* G298S MNs, TDP-43 was found in both the nucleus and the cytoplasm (Fig. 3a). We further detected subcellular distribution of TDP-43 by immunoelectron microscopy which showed that TDP-43 translocation was found in almost all TDP-43 MNs and sporadic MNs, but not WT MNs. In MNs from sporadic ALS cases (hereinafter referred to as sporadic MNs), there were less but larger clusters of gold particles, compared to those containing *TDP43* mutant (Fig. 3b). Few gold particles deposited onto the mitochondria in both *TDP-43* MNs and sporadic MNs. We did not detect SOD1 aggregates in *TDP-43* mutant and sporadic ALS MNs (data not shown). The protein aggregates might damage the protein clearance system of the cell. So we quantified the TDP43 protein level in MNs. Our results showed that there existed no significant difference in TDP43 protein level between ALS MNs and control MNs at this early stage (Fig. 3c).

### Neurofilament inclusion in MNs

**Fig. 4:**
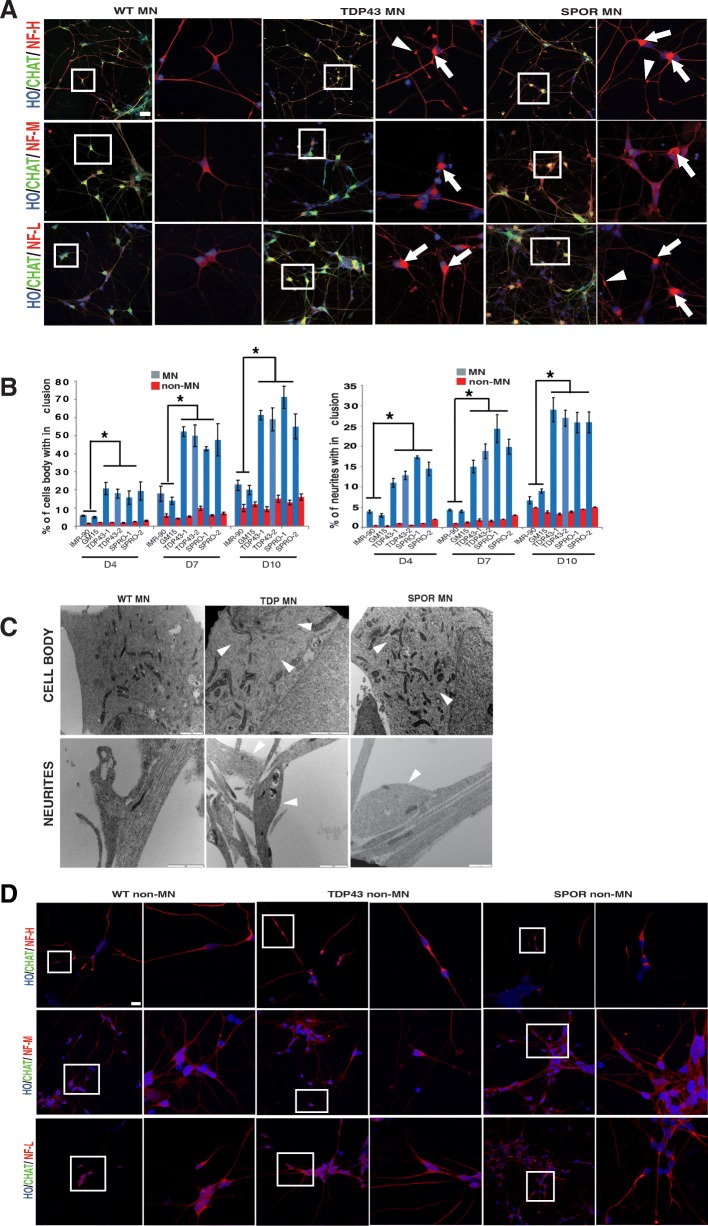
NF inclusions in ALS iPSC-derived neurons. **a** Immunofluorescent images of NF-H, NF-M, and NF-L in CHAT+ MNs. NF staining in the insets is magnified on the right panel. Arrows indicate NF inclusions in the cell body; arrowheads indicate NF inclusions in neurites. Scale bar = 50 μm. **b** Quantification of NF inclusion-containing cell bodies and neurites in MNs and non-MNs at days 4, 7, and 10 after plating. *p < 0.05. **c** EM showing NF arrangement in cell body and neuritis of MN cultures. Scale bar = 2 μm. **d** Immunofluorescent images of NF-H, NF-M, and NF-L in non-MNs. Scale bar = 50 μm

The most common and specific pathological finding in either familial or sporadic ALS is neurofilament accumulation in the perikaryon and proximal axons of spinal cord motor neurons [14]. After being stained with NF-200, NF-145, and NF-68, NF aggregates were found in these ALS MNs (Fig. 4a). The criteria for identifying NF aggregates are included in the “Materials and methods” section. The aggregates contained distinct, inclusion-like focal accumulation of immunoreactive products as previously described [10]. The aggregates were distributed in both cell body and neurites. The ratio of MNs having NF aggregation was significantly higher in ALS MNs than in WT control ones (*P* < 0.05) (Fig. 4b). When cultured for 4, 7, and 10 days, the number of NF inclusions both in cell body and on neurites increased over time (Fig. 4b). The size of the NF inclusion was 2.94~3.72 × 10^−4^ mm^2^ in cell body and 3.75–5.53 × 10^−6^ mm^2^ on neurites at D10. Electron microscopy showed that a lot of disorganized neurofilaments occupied most of the cytoplasm. Numerous proximal neurites were swelling and swirls of neurofilaments accumulated in these swollen neurite segments (Fig. 4c). At the same time, it is of interest to note that, under the same culture condition, fewer NF inclusions were found in non-MN than in motor neurons (*P* < 0.05) (Fig. 4b, d).

### Mitochondria density was low in ALS MN neurites

**Fig. 5:**
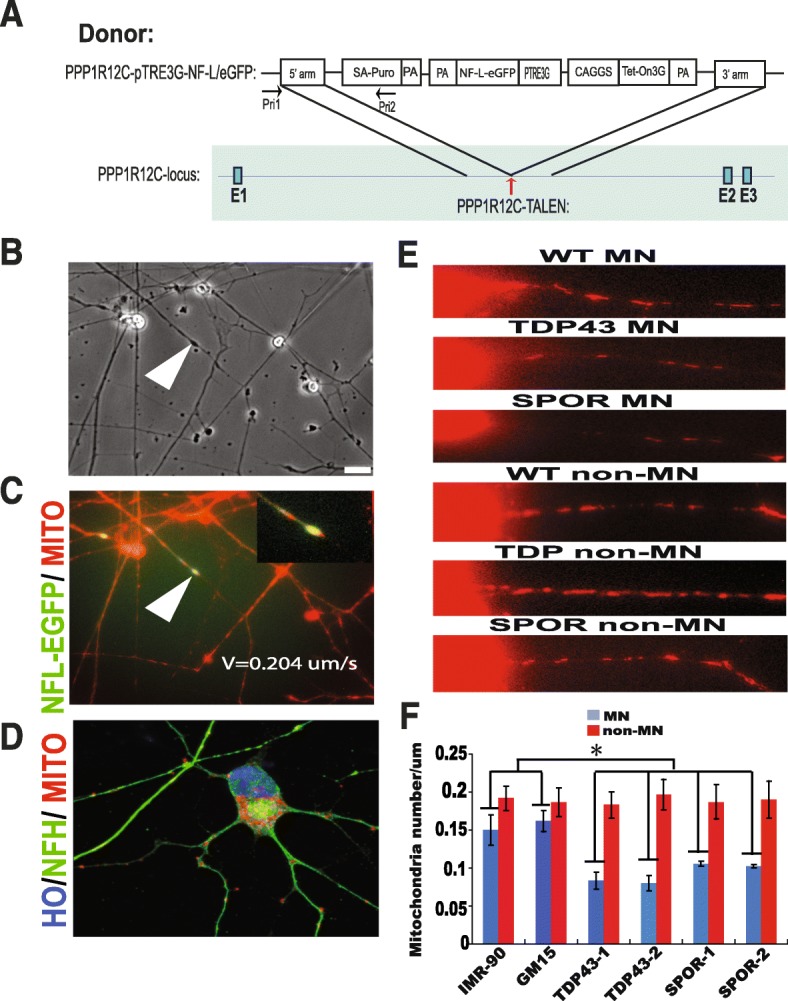
Mitochondria density decreased on the neurite in ALS MN. **a** Schematic diagram of conditional expression of NL-EGFP fusion protein in the AAVS1 sites via TALEN. **b** Phase contrast image of NF inclusion in neurites. Scale bar = 50 μm. **c** Neurofilament inclusion blocks the mitochondria movement on the neurites. Arrow head indicates colocalization of NF inclusion and mitochondria on neuritis. **d** Mitotracker staining of mitochondria shows colocalization of NF inclusions and mitochondria in cell body. **e**, **f** Mitochondria density decreased in ALS MN, but not in non-MN (*P < 0.05)

In order to directly observe the neurofilament inclusion, we inserted pTRE3G-NFL-EGFP cassette to the PPP1R12C locus by using TALEN-mediated homologous recombination in ALS iPSCs to induce the expression of NFL-EGFP fusion protein (Fig. 5a). At the same time, we labeled the mitochondria with mitotracker-ORG. Thereby, we could examine the NF-inclusion and mitochondria interaction in vivo. The video showed that some mitochondria moved into, rather than out of NF inclusion (Additional files 3, 4, 5 and 6). Then, NF and mitochondria moved at a velocity of 0.204 ± 0.035 μm/s, which confirmed directly that NF-inclusion may disrupt axonal transport of mitochondria (Fig. 5b, c).

It was reported that NF serves as a docking site for the mitochondria and regulates their spatial distribution along axons [15]. Since neurofilaments of ALS MN are impaired, we co-stained the cells using NF200 plus mitotracker-ORG. Our results showed that, within or adjacent to the NF inclusions, a large number of mitochondria accumulated (Fig. 5d). EM also showed that many mitochondria got stuck and were clustered in the NF inclusion (Fig. 4c).

Since NF-inclusion affected mitochondria movement and distribution, we determined the density of the mitochondria on a 50-μm segment from cell body of MNs. The results exhibited that the neurite mitochondria density was significantly lower in ALS MNs than in the control MNs, but the distribution of mitochondria of non-MN was not affected (Fig. 5e, f).

### Transgenesis of *TDP-43* G298S into AAVS locus in HES effected phenotype of patient-derived G289S MNs

**Fig. 6:**
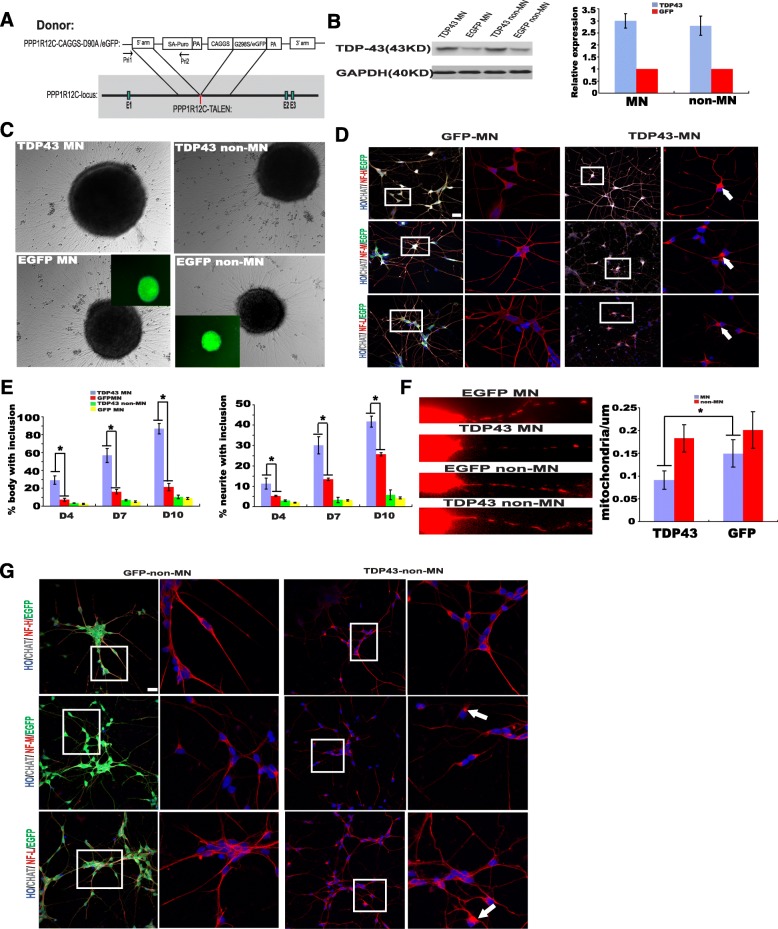
NF aggregation and mitochondria density in WT neurons expressing G298S TDP43. **a** Schematic diagram showing TALEN-mediated insertion of G298S TDP43 or EGFP into the AAVS1 locus. **b** Western blots and relative expression of TDP43 (to GPDH) in MNs and non-MN derived from hESCs expressing G298S TDP43 or EGFP. **c** Phase contrast and corresponding GFP fluorescent images of MNs and non-MNs from the transgenic hESCs. **d** Immunofluorescent images of NF-H, NF-M, and NF-L in CHAT+ cells from TDP43- and EGFP-expressing hESCs. NF staining in the insets is magnified on the right panel. Arrows indicate NF inclusions in the cell body; arrowheads indicate NF inclusions in neurites. Scale bar = 50 μm. **e** Quantification of NF inclusion-containing cell bodies (**c**) and neurites (**d**) at days 4, 7, and 10 after plating neurons. **f** Mitochondria number decreased in TDP43-expressing MNs as compared to EGFP-expressing MNs. *p < 0.05. **g** Immunofluorescent images of NF-H, NF-M, and NF-L in non-MN cells from TDP43- and EGFP-expressing hESCs. Scale bar = 50 μm

To confirm that the NF and mitochondria impairment in patient-derived G289S MNs did not result from individual and cloning differences between *TDP-43* G298S MNs and controls, but was caused by the toxicity of mutant TDP-43, we inserted the mutant cDNA into PPP1R12C-locus of human embryonic stem cells (H9) by TALEN-mediated homologous recombination (Fig. 6a). Thus, we could consistently express G298S TDP43 mutant protein in H9 and their derivates. We differentiated two embryonic stem cells lines to MNs under the condition identical to that of the patient-derived iPS cells. Western blotting showed that TDP-43 expression in *TDP43*-expressing group was about 3 times that of control group (Fig. 6b, c). It is of note that NF-inclusion increased progressively in G298S *TDP43*-expressing MNs compared to *EGFP*-expressing MNs, but not in non-MNs (Fig. 6d, e, g). Furthermore, mitochondria density in neurites was specifically decreased in G298S *TDP43*-expressing MNs (Fig. 6f).

### Vulnerability of ALS MNs to MG132 stress was increased

**Fig. 7:**
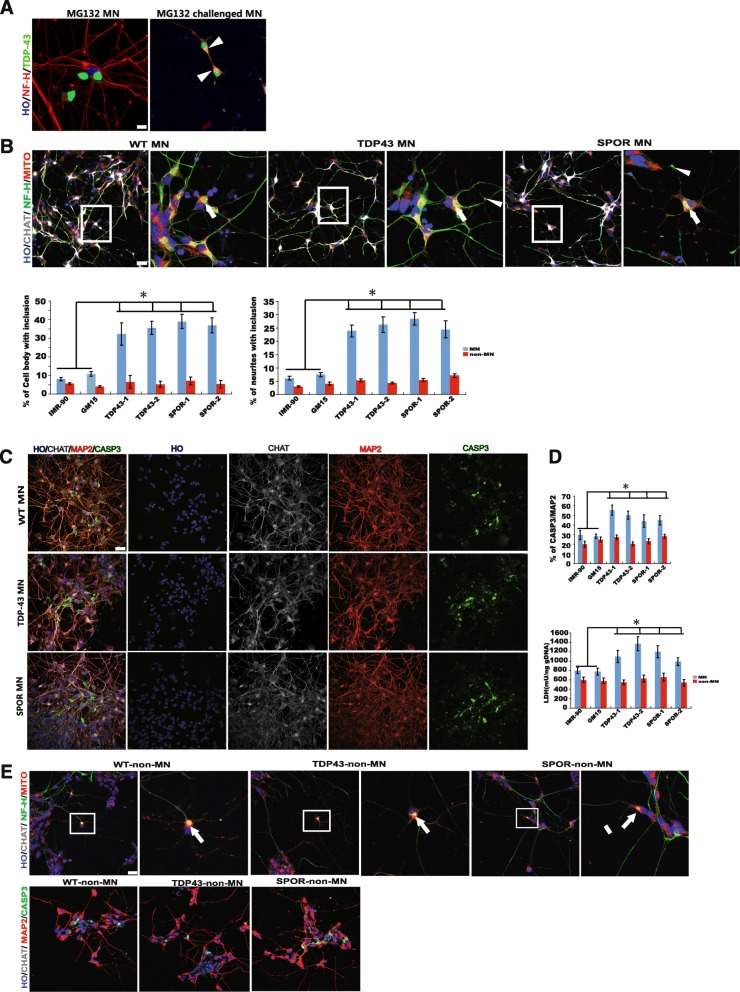
ALS MNs are vulnerable to MG132 challenge. **a** TDP43 translocation in MN after challenge with MG132. Scale bar = 20 μm. **b** NF inclusion significantly increased in ALS MNs as compared with WT MN but not in non-MNs after challenge with MG132. Scale bar = 50 μm. **c** Caspase 3-positive cells increased significantly in ALS MNs compared with WT MN but not in non-MNs after challenge with MG132. (*P < 0.05). Scale bar = 50 μm. **d** LDH concentration increased in ALS MN compared with WT MN but not non-MN after challenge with MG132. (*P < 0.05). **e** Immunofluorescent images of NF-H and Caspase3 in non-MN cells from TDP43- and EGFP-expressing hESCs after challenge with MG132. Scale bar = 50 μm

Cytoplasmic inclusions are a major pathological hallmark of ALS. One of the hypotheses is that folded proteins play a crucial role in the mediation of the neurotoxic effects [16]. MG132 is a proteasome inhibitor and reduce the degradation of ubiquitin-conjugated proteins. We wanted to know whether exposure to MG132 ALS MNs could result in a more robust phenotype. After cultured dissociated MNs for 4 days, we challenged the cell using 0.6 μM MG132 for 24 h. Figure 7a shows that the challenge led to TDP43 translocation even in WT MNs. However, more ALS MNs had NF inclusion in neurites and cell body compared to controls. Many mitochondria are caught in NF inclusion. But there is no difference in the ratio of NF inclusion between ALS non-MNs and controls (Fig. 7b, e). Finally, to find out if the ALS MNs are prone to death after challenge, we measured the concentration of LDH in the medium and quantified the cleaved caspase3-positive cell by immunostaining. It was found that LDH level was relatively higher in the medium of ALS MNs. There were less cleaved caspase3-positive cells in the controls than in MNs. Our data suggested that ALS MNs are more vulnerable compared to the WT MNs (Fig. 7c, d).

## Discussion

ALS patients begin to present with symptoms during adulthood [17]. Therefore, mutant TARDBP MNs or sporadic MNs could be induced with the same efficiency with WT cell lines by employing ALS patient-derived iPSCs, although it was reported that some phenotypic alterations, accompanied by some subtle behavioral abnormalities might take place during early stage of ALS [18].

One challenge in establishing stem cell models is to enrich target cells, such as MNs. In this study, we developed a technique that could differentiate multipotent cells into MNs, with up to 90% of the cells being able to express MNX1 and CHAT. The protocol used three small molecules to induce neuroepithelial differentiation as previously reported [19–21]. In particular, CHIR99021, a GSK3 beta inhibitor, activated WNT signaling, increased the neural differentiation, and synchronized the primitive neuroepithelia for subsequent patterning. Spinal cord non-MNs were also generated by inhibiting hedgehog signaling with cyclopamine. Compared to previous methods, because most neurodegenerative phenotypes are found in mature neurons, we used a notch inhibitor to synchronize the production of mature MNs and prevent later-born neurons to generate homogenous mature MNs [21, 22].

TDP-43 is prone to aggregation. Accumulated or mutant TDP-43-induced neurodegenerative diseases [23]. ALS-linked mutations increase its toxicity, tendency to aggregate, and mislocalization to the cytoplasm [4]. Both sporadic and familial ALS are characterized pathologically by ubiquitinated TDP-43 inclusions in the surviving spinal MNs [24]. Inconsistent with another report [25], our immunostaining showed that TDP-43 aggregation was not obvious in these human MNs. On the other hand, under immunoelectron microscope, small aggregates did exist in sporadic MNs. Nonetheless, in *TDP-43* G298S MNs, TDP-43 aggregates were smaller compared to sporadic ALS, but translocation was more conspicuous. Our data indicated that our stem cell method could mimic TDP-43 protein pathology even when the MNs were very “young”. Our results also indicated that toxicity of misfolded TDP-43 inclusions or aberrant RNA processing because of translocation of TDP-43 represents an early step in the molecular cascade tied to MN degeneration.

It has been proposed that extreme levels of mutant protein in mouse models can produce artifact pathology [26, 27]. So we first examined the TDP43 expression in these “young” MNs, but we did not observed any over-expression of TDP43 in ALS MNs. The ubiquitin-proteasome system (UPS) in ALS vulnerable tissues was reportedly not working properly [28]. But our data suggested that the altered UPS system was likely to be a secondary event. Possibly, at the early stage, the intracellular proteolysis system can compensate well and can maintain the balance of TDP43 expression.

Neurofilament accumulation in the perikaryon and proximal axons of spinal cord motor neurons is the most common and an early hallmark of both familial and sporadic ALS [29]. In our study, immunostaining clearly exhibited that G298S *TDP43* MNs and sporadic MNs tend to form NF inclusions as early as day 4 after MN plating as compared to WT MNs. Moreover, the NF inclusions were further confirmed by EM. It is of interest that non-MNs possess fewer cytoplasm and NF inclusions. NF is the most abundant fibrillar components of the axon. NF works together with microtubules and microfilaments to enhance structural integrity, maintain cell morphology, and promote organelle motility [30]. Transgenic mouse studies demonstrated that over-expression of normal and mutant NF proteins can provoke a MN pathology characterized by the presence of abnormal NF accumulations resembling those found in ALS [31]. But how NF inclusions contribute to human diseases remains unknown. By live imaging, immunostaining, and EM, we readily observed that NF inclusions disrupt mitochondrial movement/distribution and decrease the mitochondrial density in neurites. Proper density and localization of mitochondria are of critical importance for the maintenance of synapses. Neurons are very sensitive to perturbations in terms of mitochondrial distribution [32]. Synaptic loss occurs long before neuronal death in ALS patients [33]. In conclusion, our data suggested that abnormal mitochondrial distribution caused by NF inclusion might be one of common pathological mechanisms that lead to selective MN vulnerability in both sporadic and family ALS. Another study also reveals a strong association between mitochondrial functions and neurodegeneration in human iPS-derived motor neurons from sporadic ALS patients [34].

Individual variations and even differences among individual stem cell clones pose great obstacles to the establishment of a disease model by using human stem cells. To address this barrier and establish cause-effect relationships between G298S *TDP-43* mutation and aberrant phenotypes of MNs, we edited genome with transcription activator-like effector nucleases (TALENs) technology to integrate the same mutation into a safe harbor gene locus (PPP1R12C, also known as AAVS1) in the genome of human embryonic stem cells (H9). Until now, it is believed that nuclear depletion, cytoplasmic accumulation of insoluble TDP-43, or both of them may contribute to the onset and progression of ALS. However, no definite conclusion has been reached [35]. But our data suggested that mutant *TDP-43* caused abnormal MN phenotype by gaining toxicity of TDP-43.

To confirm the phenotype obtained from the stem cell model and obtain prominent phenotypes, we further challenged MNs with a proteasome inhibitor, MG132. Our data suggested that G298S *TDP-43* and sporadic MNs were more vulnerable to perturbation. This vulnerability was specific for MNs, with some accompanying phenotype changes, such as NF inclusion, mitochondria distribution impairment, and activation of CASP3.

## Conclusion

In summary, we established an enriched MN platform which can model the hallmark pathology of ALS. We found that TDP43 protein pathology appeared early in ALS spinal MNs, which is consistent with other studies. For the first time, our data proved that NF inclusion and impairment of mitochondria distribution are early and common pathology in both family and sporadic ALS. The pathology subjects MNs to the huge risk of degeneration triggered by environmental factors. These findings will help us gain insight into pathogenesis of the condition and screen relevant drugs for the disease.

## Additional files

Additional file 1: **Table S1.** (DOCX 13 kb)

Additional file 2: **Table S2.** (DOCX 13 kb)

Additional file 3: **Video S1.** NF-LGFP40X_7_ch1.AVI. NF inclusions in NF-L-GFP merge MN neurites in light microscope (× 40). (AVI 15305 kb)

Additional file 4: **Video S2.** NF-LGFP40X_7_ch2.AVI. NF inclusions in NF-L-GFP merge MN neurites in green channel. (AVI 15305 kb)

Additional file 5: **Video S3.** NF-LGFP40X_7_ch3.AVI. Mitochondrial movement in NF-L-GFP merge MN neurites in red channel. (AVI 15305 kb)

Additional file 6: **Video S4.** NF-LGFP40X_7_ch4.AVI. Some mitochondria moved into, rather out of NF inclusions. (AVI 45903 kb)

## Abbreviations

ALS: Amyotrophic lateral sclerosis
ESC: Embryonic stem cells
iPSC: Induced pluripotent cells
MN: Motor neuron
NF: Neurofilament
SCID: Severe combined immunodeficient
TDP-43: TAR DNA-binding protein 43
WT: Wild type
