# Correction to: Modeling hallmark pathology using motor neurons derived from the family and sporadic amyotrophic lateral sclerosis patient-specific iPS cells

**DOI:** 10.1186/s13287-019-1211-3

**Published:** 2019-03-15

**Authors:** Xuejiao Sun, Jianyuan Song, Hailong Huang, Hong Chen, Kun Qian

**Affiliations:** 1 Department of Rehabilitation Medicine, Tongji Hospital, Tongji Medical College, Huazhong University of Science and Technology, Jiefang Avenue 1095, Wuhan, 430030 China; 2 Reproductive Medicine Center, Tongji Hospital, Tongji Medicine College, Huazhong University of Science and Technology, Jiefang Avenue 1095, Wuhan, 430030 China

**Correction to: Stem Cell Res Ther**

**https://doi.org/10.1186/s13287-018-1048-1**

The original article [1] was submitted and published without co-author Hong Chen’s permission. The author, Hong Chen has therefore requested to have her name disregarded from the author list. All of the authors agree with this change.
