# Supplementary material for: Modeling hallmark pathology using motor neurons derived from the family and sporadic amyotrophic lateral sclerosis patient-specific iPS cells
Source: Stem Cell Res Ther. 2018 Nov 15;9:315. doi: 10.1186/s13287-018-1048-1 (PMC6238404; doi:10.1186/s13287-018-1048-1)
Supplement: Supplementary file 1 — Table S1. (DOCX 13 kb) [file 13287_2018_1048_MOESM1_ESM.docx]

**Table S1.** Human pluripotent stem cell lines and replications in experimental procedures

| Code | Diagnosis | Gender | Age at biopsy | clones | Replications |
| --- | --- | --- | --- | --- | --- |
| GM15 | Normal control | male | 61 | 1 | 3 |
| TDP(G298S) | Family ALS | male | 64 | 2 | 3 |
| SPORADIC-1 | Sporadic ALS | male | 59 | 1 | 3 |
| SPORADIC-2 | Sporadic ALS | male | 72 | 1 | 3 |
