# Supplementary material for: Modeling hallmark pathology using motor neurons derived from the family and sporadic amyotrophic lateral sclerosis patient-specific iPS cells
Source: Stem Cell Res Ther. 2018 Nov 15;9:315. doi: 10.1186/s13287-018-1048-1 (PMC6238404; doi:10.1186/s13287-018-1048-1)
Supplement: Supplementary file 2 — Table S2. (DOCX 13 kb) [file 13287_2018_1048_MOESM2_ESM.docx]

**Table S2.** **List of antibodies in experimental procedures**

| Antibody | Isotype | Dilution | Source |
| --- | --- | --- | --- |
| MNX1/HB9 | Mouse IgG | 1:50 | DSHB |
| OLIG2 | Rabbit IgG | 1:500 | Chemicon& Millipore |
| CHAT | Goat IgG | 1:300 | Chemicon& Millipore |
| TUJ1 | Mouse IgG | 1:200 | Chemicon& Millipore |
| Neurofilament-200 | Rabbit IgG | 1:2000 | Sigma |
| Neurofilament-145 | Mouse IgG | 1:500 | Chemicon& Millipore |
| Neurofilament-68 | Mouse IgG | 1:200 | Sigma |
| TARDBP | Mouse IgG | 1:400 | Proteintech Group |
| Cleaved capase3 | Rabbit IgG | 1:500 | Cell signaling |
